# Under the humble mask: Investigating when and how leader-expressed humility leads to employee voice

**DOI:** 10.3389/fpsyg.2022.950059

**Published:** 2022-08-22

**Authors:** Wen-Qian Zou, Shu-Chen Chen

**Affiliations:** ^1^Ningbo Childhood Education College, Ningbo, China; ^2^Department of Business Administration, Ming Chuan University, Taipei, Taiwan

**Keywords:** leader-expressed humility, perceived manipulative intention, psychological safety, self-efficacy, voice

## Abstract

This study aimed to explore the psychological mechanisms through which psychological safety and self-efficacy mediate the relationship between leader-expressed humility and employee voice. Moreover, attribution theory was applied to examine the possible detrimental effects of leader-expressed humility when employees perceive manipulative intentions in their humble leader. The current study proposed the leader’s manipulative intention as a moderator to weaken the indirect relationships between leader-expressed humility and employee voice through psychological safety and self-efficacy. Time-lagged supervisor–subordinate matched data were used to test the model. Our findings reveal leader’s manipulative intention weakens the positive effect that leader-expressed humility impacts on employee voice through psychological safety and self-efficacy. The implications of the findings were discussed from both theoretical and practical perspectives.

## Introduction

Leader humility is a topic that has developed from bottom-up leadership (Owens and Hekman, 2012, 2016; Owens et al., 2013; Ou et al., 2014). Expressed humility can be defined as an interpersonal characteristic that manifests as a willingness to view oneself accurately, an appreciation of others’ strengths, and teachability based on Owens’s (2013) framework of expressed humility in organizations. When leaders express humility, revealing their human fallibility, and are willing to accept suggestions and implement new ideas, it signals to their employees that expressing themselves is effective and worthwhile (Li et al., 2018). Although studies have begun to demonstrated the relationship between leader-expressed humility and employee voice (Jeung and Yoon, 2018; Bharanitharan et al., 2019), few studies have explored the psychological mechanisms explaining how leader-expressed humility influences employee voice behavior. To address this research gap, this study adopted social information process theory (SIP theory; Salancik and Pfeffer, 1978) to explain how the workplace-related social cues of leader-expressed humility influence employees to adjust their attitude and behavior. Voice behaviors are target-sensitive and stimulate changes in the manner of doing things (Liu et al., 2010). People are unwilling to speak up unless they feel safe in social interaction contexts. Moreover, voice stimulates changes through constructive suggestions, and people must identify work-related problems and propose feasible suggestions. Employees can engage in voice behaviors when they believe in their ability to propose constructive suggestions. Humble leaders exhibit openness to new information and are more understanding of subordinates’ mistakes at work (Owens and Hekman, 2012; Owens et al., 2013). Drawing on SIP, these humble behaviors signal to employees that: “It is safe to express myself,” thus increases employees’ psychological safety (Nembhard and Edmondson, 2006). Leaders who express humility are willing to acknowledge and appreciate employees’ contributions. These ongoing verbal encouragements and behavioral supports send a “I can do it” signal to employees which can increases employees’ self-efficacy (Wei et al., 2015; Lee et al., 2017). Therefore, the present study aimed to explore the impacts of leader-expressed humility on employees’ voice through psychological safety and self-efficacy.

To date, most research has focused on the positive effects of leader-expressed humility.

However, humility may affect in negative ways as well (Owens and Hekman, 2012). In recent years, political humility has been a topic of research (D’Errico and Poggi, 2019; D’Errico et al., 2019), studies have demonstrated that humble communication elicited negative emotions and evaluations of the politician (D’Errico, 2019). Thus, to comprehensively understand the impact of humility, the boundary conditions for humility should be properly examined (Owens et al., 2015; Wang et al., 2018). The study of functional perspectives has revealed that leaders may vary their behaviors situationally (Liu et al., 2017). As leader-expressed humility is measured behaviorally, research focuses on a pattern of displayed behaviors that emerges in interpersonal interactions and is readily apparent to others (Owens et al., 2013). It is difficult to determine whether a person really has an inner state of motivation only by displayed behaviors (Wright et al., 2018), a non-humble leader can counterdispositionally express humility to reach desired goals without actually being humble (Yang et al., 2019). We suggest that employees are likely to assess leader-expressed humility as either genuine or hypocritical. Although leaders may publicly exhibit humility in order to gain common goods, in private they may be driven by self-interest. If employees perceive that their leader is behaving humbly to reach their own desired goals without actually being humble, would such leader-expressed humility with a hidden agenda also positively affect employees’ behaviors? To answer this question, we used attribution theory to explore the possible negative effects of leader-expressed humble behaviors when these behaviors are perceived as manipulative by their employees. According to attribution theory (Kelley, 1973), an employee’s attribution of the intention underlying a leader’s specific behavior affects the employee’s subsequent response (Dasborough and Ashkanasy, 2002; Martinko et al., 2011; Chernyak-Hai and Tziner, 2021). Dasborough and Ashkanasy (2002) indicated that perceived manipulative intention is a useful tool for employees to evaluate the supervisors’ behaviors, where perceived manipulative intention refers to the perception of how much an individual’s action is driven by self-interest. When employees interpret leader-expressed humility as a manipulative effort to benefit himself or herself, they are likely to label the leader as pseudo humble. In turn, employees express negative attitudes and behavioral responses toward the leader (Dasborough and Ashkanasy, 2004; Lin et al., 2017). Thus, we argue the positive effect of leader-expressed humility on employee voice through psychological safety and self-efficacy are more likely to be reduced when the employees attribute their leader’s expressed humility to manipulative intention.

**FIGURE 1:**
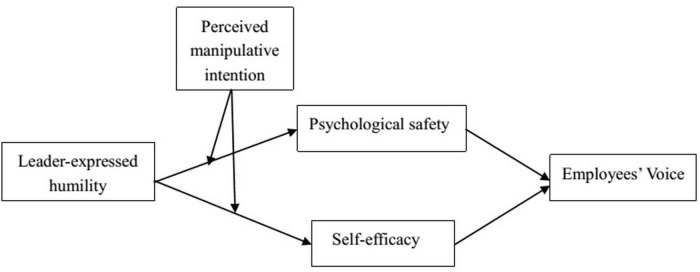
Theoretical model.

The main contributions of this study to the literature on leader humility and leadership are threefold. First, Morrison (2011, 2014) indicated that employee voice in an organization is affected by efficacy and safety. Studies on leadership and voice have been mostly based on only one of these factors (Detert and Burris, 2007; Walumbwa and Schaubroeck, 2009; Wang et al., 2015). In terms of employee psychology, self-efficacy is defined as the employees’ confidence in their ability to engage in voice behavior, whereas psychological safety refers to the individuals’ perception that the environment is a safe one in which to take interpersonal risks (Edmondson, 1999). By combining psychological safety and self-efficacy, the present study elucidated the psychological mechanisms affecting the relationship between leader humility and employee voice. Second, Owens and Hekman (2012) indicated that leader humility is effective only if employees perceive their leader to be sincere and authentic. However, studies have not yet verified the boundary conditions for leader humility. The present study aims to explore whether the leader’s manipulative intention moderate the relationship between the leader’s expression of humility and voice behavior, which in turn is mediated by employees’ psychological safety and self-efficacy? Finally, by relating attributions to leader-expressed humility, we also respond to the Martinko et al. (2007, 2011) and Martinko and Mackey (2019) call for paying more attention to attribution theory in the organizational sciences. Our theoretical model is shown in Figure 1.

## Theory and hypotheses

### Leader-expressed humility and voice

Expressed humility focuses on the expressed, interpersonal nature of humility and is manifested through power-equalizing behaviors (Ou et al., 2014; Owens and Hekman, 2016). Owens and Hekman (2012) generalized three interpersonal characteristics of leader-expressed humility. The first characteristic is a willingness to show their “humanity” to employees, which enables leaders to admit to their mistakes and limitations and willingly assume responsibility for mistakes and failures. The second characteristic is an appreciation of others’ strengths, which enables leaders to discover and appreciate the strengths and contributions of employees. The third characteristic is teachability, which enables leaders to seek improvements, and engage in ongoing learning. In contrast to the top-down approach of charismatic and proactive leadership, leader-expressed humility is characterized by a shift in attention from leaders to employees (Owens and Hekman, 2016).

We argue that leader-expressed humility is positively related to employees’ voice behavior. Leaders who express humility display human fallibility and acknowledge their limitations and weaknesses. In acknowledgment of their own weaknesses, humble leaders may sometimes ask employees to help them remedy or compensate for these weaknesses (Owens and Hekman, 2012). When humble leaders undertake a task they do not excel at, they actively seek help and advice from employees, thus promoting employee voice. Moreover, humble leaders express appreciation and respect for employees’ strengths and contributions. As these displays allow employees to demonstrate their superiority and enhance their self-perceived status, employees are motivated to engage in voice behavior further (Janssen and Gao, 2015). Third, humble leaders who exhibit teachability engage in ongoing learning, have a habit of listening, and are often receptive to new knowledge and others’ ideas (Owens and Hekman, 2012; Li et al., 2018). All the aforementioned behaviors of humble leaders signal that they (as leaders) are willing to listen to their employees’ opinions. Hence, we hypothesize the following.

*Hypothesis 1:* Leader-expressed humility is positively related to voice behavior.

### Psychological safety and self-efficacy as mediators

Voice behavior is both target-sensitive and risky. As a result, most employees consider challenging managers too great a risk. Some employees may perceive that speaking up in their organization puts them with negative labels, such as “troublemaker,” “disobedient member,” or “disrespectful employee” (Milliken et al., 2003). Employees are sensitive to the informational signals transmitted by leaders because of leaders’ higher status (Chiu et al., 2016). People are unwilling to speak up unless they feel safe in social interaction contexts (Xu et al., 2019). Psychological safety refers to the shared belief that interpersonal risk taking is safe in a given environment (Kahn, 1990; Edmondson, 1999). It refers to the employees’ perceptions of being able to ask questions, seek feedback, report mistakes, and propose new ideas without fear of incurring negative consequences in their self-image, status, or career development (Edmondson et al., 2004). Humble leaders show openness to their limitations and mistakes, and they actively seek others’ new ideas or feedback, even if it is critical (Owens et al., 2013). Additionally, humble leaders allow their employees to realize that making mistakes is normal and that accepting mistakes contributes to personal growth rather than being a source of blame (Owens and Hekman, 2012, 2016; Bharanitharan et al., 2020). Drawing on SIP (Salancik and Pfeffer, 1978), these expressions of humility send a signal to employees that: “It’s safe to express myself even if making mistakes,” thus increases employees’ psychological safety. Humble leaders often express their appreciation and respect for the efforts and contributions of their employees. As a result, employees believe that they work in a respectful environment, and that they can express their opinions without fear of a negative reaction from their leaders (Qian et al., 2020). Taken together, these findings suggest that employees tend to feel psychologically safer and perceive a lower risk of speaking up when under a humble leader, which in turn increases their voice behavior (Detert and Burris, 2007; Walumbwa and Schaubroeck, 2009). Thus, we hypothesize the following.

*Hypothesis 2:* Psychological safety mediates the relationship between leader-expressed humility and voice behavior.

Moreover, we argue that leader-expressed humility triggers more voice behavior through increased employees’ self-efficacy. As it reflects individual cognition toward the true self (Frostenson, 2016), leader-expressed humility nurtures and promotes self-recognition in employees, thereby enhancing their concept of self (Owens and Hekman, 2012). Leader-expressed humility manifests through a set of power-equalizing behaviors (Ou et al., 2014; Owens and Hekman, 2016). Such power-equalizing behaviors allow bureaucratic constraints to be removed, so that their employees are able to feel released and enjoy a sense of power at work (Jeung and Yoon, 2018), which helps employees recognize their significance, and then increases employees’ self-efficacy (Ju et al., 2019). Moreover, humble leaders frequently express appreciation for the unique abilities and contributions of their employees, these ongoing verbal encouragements and behavioral supports from humble leaders enhance employees’ confidence in their ability (Hu et al., 2018), and send a signal to employees that: “I can do it,” which in turn increases employees’ self-efficacy. Employees experience increased self-efficacy, which causes them to realize that they possess the necessary knowledge to engage in proactive behavior (Judge and Bono, 2001). Employees with high self-efficacy believe that they can perform well in their organization (Bandura, 1977), and adopt proactive strategies and transform their thoughts into constructive actions, thus increasing voice behavior (Beltrán-Martín et al., 2017). Thus, we hypothesize the following.

*Hypothesis 3:* Self-efficacy mediates the relationship between leader-expressed humility and voice behavior.

### Perceived manipulative intention as a moderator

Studies demonstrate that it is likely to be inconsistent between individuals’ inner motivation and externally displayed behaviors (Wright et al., 2018; Bharanitharan et al., 2020). Leaders can express humility instrumentally to reach desired goals without actually being humble (Yang et al., 2019). Because of behaviors exhibited by authentic and pseudo leaders may be similar, researchers suggest the leader’s manipulative intentions (the degree of words and actions motivated by self-interest) are vital in distinguishing them (Dasborough and Ashkanasy, 2002; Lin et al., 2017). Attribution is an individual’s perception of the reasons behind their own or others’ behavior (Kelley, 1973; Weiner, 1985). In the attribution process, intention is a vital triggering element that helps the perceivers in their evaluation, interpretation, and assigning of meaning to their observations (Ferris et al., 1995). Drawing on attribution theory, the employee’s attribution of leader’s intention determines how they view the leader’s behavior, which in turn determines their reactions (Eberly and Fong, 2013; Chernyak-Hai and Tziner, 2021). Studies on leadership have suggested that employees’ subjective attributions regarding the self-interested motives of their leaders are critical to gaining an understanding of employees’ reactions (Ou et al., 2014). For example, Sue-Chan et al. (2011) demonstrated that when employees attribute their leader’s coaching behavior to self-interest, this coaching negatively influences employees’ job performance. Lin et al. (2017) found that when employees attribute their leader’s transformational behavior to manipulative intention, the positive effect of transformational leadership is weakened. When a leader’s behavior is attributed to manipulative intentions (Dasborough and Ashkanasy, 2004), employees are more likely to believe their leaders are behaving insincerely just for their own benefit. Employees tend to label the leader-expressed humility as pseudo humble when they perceive leaders’ manipulative intention (Eberly and Fong, 2013; Bharanitharan et al., 2020), and this will result in negative effects (Dasborough and Ashkanasy, 2002).

As reported by Cha and Edmondson (2006), pseudo perceptions frequently produce disenchantment, which entails anger, disappointment, and a loss of trust. Empirical research further revealed that pseudo leadership positively relates to fear and perceptions of job insecurity among employees (Christie et al., 2011). When employees attribute leader-expressed humility to manipulative intentions, they consider their leader’s expressions of humility as intended only to manipulate for personal gain. Employees may associate manipulative attribution with leader-expressed humility and be likely to reduce their trust and feel uncertain, which reduces their psychological safety. Leader humility advocates other enhancements through their openness in soliciting feedback (Owens et al., 2015). Arguably, there is a difference between manipulative leaders who display appreciation and ask for feedback and leaders who acknowledge and appreciate employees’ contributions with no manipulative intent. If employees attribute such humble behaviors to manipulative intention, then these expressed humble behaviors will not enhance self-efficacy because employees believe that they are done under false pretenses. Based on these arguments, this study proposes the relationship between leader-expressed humility and employees’ psychological safety and self-efficacy will be weakened when employees attribute leader-expressed humility to higher manipulative intention. Hence, we hypothesize the following.

*Hypothesis 4:* Perceived manipulative intention moderates the relationship between leader-expressed humility and psychological safety, such that the positive relationship is weaker when perceived manipulative intention is high.

*Hypothesis 5:* Perceived manipulative intention moderates the relationship between leader-expressed humility and self-efficacy, such that the positive relationship is weaker when perceived manipulative intention is high.

### Integrated model

When employees attribute leader-expressed humble behaviors to manipulative intention, the leader’s behavior is perceived as selfish, disingenuous and driven by an ulterior motive, which in turn to feel psychologically unsafe when interacting with their pseudo leader. Thus, to avoid the risks of speaking up, employees choose to minimize voice behavior. Similarly, when employees attribute a leader’s humble behavior to manipulative intention, employees tend to doubt the authenticity of their leader’s appreciation and verbal encouragement. As deceptive verbal persuasion do negatively influences individual self-efficacy, thereby reducing employees’ voice behavior. Hence, we hypothesize the following.

*Hypothesis 6:* Perceived manipulative intention moderates the indirect effect of leader-expressed humility on voice behavior through psychological safety, such that the indirect effect is weaker when perceived manipulative intention is high.

*Hypothesis 7:* Perceived manipulative intention moderates the indirect effect of leader-expressed humility on voice behavior through self-efficacy, such that the indirect effect is weaker when perceived manipulative intention is high.

## Materials and methods

### Participants and procedure

As humility leadership may exist in any work involving leader-employee dyad (Shaw and Mao, 2021), the dyadic data in this study were collected from 31 organizations in southeastern China from diverse industries (such as education, health, technology, banking, government agencies), we first contacted the organizations’ directors and explained the objectives of the study, and then ask them to help us to select randomly one or two groups that work together and frequently communicate with each other in daily work to distribute the questionnaires (Wang et al., 2018). To reduce common method variance, we obtained multi-wave and multisource data. At Time 1, 330 employees from 46 groups received questionnaires to evaluate leader-expressed humility and perceived manipulative intention. At Time 2 (2 weeks after Time 1), two sets of questionnaires were administered separately: one for employees and a second for their group leaders. We provided 317 employees who completed Time 1 survey with questionnaire to evaluate psychological safety and self-efficacy, while 46 group leaders received a questionnaire on the voice behaviors of their employees. A researcher-assigned identification number was used to code each questionnaire in order to match employees’ responses with evaluations by their group leaders. The respondents were instructed to seal the completed questionnaires in the envelopes and return them to the researchers directly. Each respondent was offered a gift worth approximately US $3.00 upon completion.

After excluding incomplete questionnaires, we obtained 238 employees’ questionnaires matched with 45 leaders (overall return rate: 72%). Descriptive statistics for the sample are provided in Table 1. A total of 71% of the leaders were women, the average age was 42.42 years (*SD* = 5.50), and their average tenure was 11.74 years. Among the employees, 57% were women, the average age was 29.45 years (*SD* = 7.36), the average tenure was 4.97 years, and the average dyadic tenure was 3.21 years (*SD* = 3.00).

**TABLE 1:** Basic information of the sample.

Project	Types	Number of people	Proportion	N
Employees’ gender	Male	102	42.68%	238
	Female	136	57.14%	
Employees’ age	≤ 25	84	35.29%	238
	26–40 years old	142	59.66%	
	41–50 years old	9	3.78%	
	≥ 51	3	1.27%	
Employees’ industry	Education	55	23.11%	
	Health	44	18.49%	
	Technology	42	17.65%	238
	Banking	50	21.01%	
	Government agencies	47	19.74%	
Leaders’ gender	Male	13	28.89%	45
	Female	32	71.11%	
Leaders’ age	≤ 25	0	0%	45
	26–40 years old	19	42.22%	
	41–50 years old	23	51.11%	
	≥ 51	3	6.67%	
Dyadic tenure	≤ 1	80	33.61%	238
	1–3 Years	87	36.55%	
	4–10 years	57	23.95%	
	≥ 10	14	5.89%	

### Measures

The scales used in this study were translated and back-translated in order to verify the accuracy of the scales and ensure that respondents understood the questions asked in the survey (Brislin, 1986). Seven-point scales were used to rate the described measures (1: *strongly disagree*; 7: *strongly agree*).

### Leader-expressed humility

Leader-expressed humility was measured with a 9-item scale of Owens et al. (2013). Sample items included, “My leader admits it when he or she doesn’t know how to do something.” “My leader takes notice of others’ strengths.” and “My leader is willing to learn from others” (α = 0.93).

### Perceived manipulative intention

Perceived manipulative intention was measured with a 3-item scale of Dasborough and Ashkanasy (2004). Sample items included, “My leader acts in a self-serving manner.” “My leader behaves on the basis of beliefs about potential rewards he or she may gain.” and “My leader manipulates me” (α = 0.87).

#### Voice

Voice was measured with a 9-item scale of Liu et al. (2010). Sample items included, “This person speaks up to influence the leader regarding issues affecting the organization.” and “This person gives constructive suggestions to the supervisor to improve the supervisor’s work” (α = 0.95).

#### Self-efficacy

Self-efficacy was measured with a 7-item scale of Chen et al. (2001). Sample items included, “I am confident that I can perform effectively on a wide range of tasks.” and “Compared to other people, I can do most tasks very well” (α = 0.89).

#### Psychological safety

Psychological safety was measured with a 7-item scale of Tynan (2005). Sample items included, “My leader respects my abilities.” and “My leader really cares about me” (α = 0.91).

#### Control variables

Because of the possible impact of demographic variables on employees’ work attitudes and performance (Lam et al., 2015), we controlled for the gender, age, and dyadic tenure of both leaders and employees (reported by the employees).

#### Analytical strategy

Data obtained in this study were analyzed using SPSS19.0, AMOS21.0, and Mplus 7.2. Firstly, confirmatory factor analysis was performed using Amos 21.0 to determine the discriminant validity of the variables. Secondly, SPSS19.0 was used to present the descriptive statistics and correlations of all the variables. Finally, the hypotheses were tested using Mplus 7.2. Each employee’s voice behavior was rated by their respective supervisor while nested in 35 teams, and each participant’s data were nested within a supervisory unit together with that of other participants. The “Cluster” and “Type = Complex” Mplus syntax were used to account for non-independence due to the clustering of individuals within supervisory groups (Schaubroeck et al., 2017). To test the mediation hypotheses, we followed the Monte Carlo method to estimate the confidence intervals for indirect effects (Preacher and Selig, 2012).

## Results

### Confirmatory factor analysis

We compared the hypothesized five-factor model (leader-expressed humility, perceived manipulative intention, psychological safety, self-efficacy, and voice) with several alternative models (Table 2). The findings showed that among these models, the five-factor model is a significant improvement compared to the other models (χ^2^ = 1176.93, df = 582, χ^2^/df = 2.02, CFI = 0.91, TLI = 0.91, RMSEA = 0.07). Table 3 presents the descriptive statistics and correlations of all the variables.

**TABLE 2:** Confirmatory factor analysis of variables.

Model	Factors	χ^2^	d*f*	χ^2^/d*f*	CFI	TLI	RMSEA	Δχ^2^	Δd*f*
1	5-Factor: LH; PS; SE; VO; MI	1176.93	582	2.02	0.91	0.91	0.07		
2	4-Factor; LH; PS + SE; VO; MI	1888.87	587	3.22	0.81	0.79	0.10	711.94***	5
3	3-Factor; LH + MI; PS + SE; VO	2060.81	590	3.49	0.78	0.77	0.10	883.88***	8
4	2-Factor; LH + VO + MI; PS + SE	3763.24	592	6.36	0.53	0.50	0.15	2586.31***	10
5	1-Factor; LH + PS + SE + VO + MI	4498.07	594	7.57	0.42	0.39	0.17	3321.14***	12

LH, Leader-expressed humility; PS, Psychological safety; SE, Self-efficacy; MI, Manipulative intention; VO, Voice.

CFI, comparative fit index; TLI, Tucker–Lewis index; RMSEA, root mean square error of approximation.

***p < 0.001.

**TABLE 3:** Means, standard deviations and correlations (*N* = 238).

	1	2	3	4	5	6	7	8	9	10
1. Leaders’ age										
2. Leaders’ gender	0.17*									
3. Employees’ age	0.06	–0.27**								
4. Employees’ gender	0.01	0.10	–0.07							
5. Dyadic tenure	0.28**	0.23**	0.39**	–0.05						
6. Leader-expressed humility	0.13*	0.27**	–0.06	0.08	0.00	*(0.78)*				
7. Self-efficacy	0.10	0.10	0.05	0.07	0.02	0.39**	*(0.71)*			
8. Psychological safety	0.05	0.23**	–0.05	0.03	0.03	0.60**	0.40**	*(0.81)*		
9. Voice	–0.03	0.12	0.18**	0.05	0.13*	0.15*	0.22**	0.27**	*(0.83)*	
10. manipulative intention	–0.13*	–0.10	0.05	–0.02	0.08	–0.44**	–0.31**	–0.56**	–0.22**	*(0.84)*
*Mean*	42.42	1.73	29.45	0.57	3.21	4.74	4.48	4.67	3.50	1.78
*SD*	5.50	0.45	7.36	0.50	3.00	0.88	0.65	0.92	1.06	0.98

Values in parentheses represent square roots of AVE. Gender was coded 1: woman, 0: man, *p < 0.05, **p < 0.01.

### Hypotheses testing

Model 1 results (Table 4) showed that leader-expressed humility was positively related to voice behavior (β = *0.15, p* < 0.05). Thus, Hypothesis 1 was supported.

**TABLE 4:** Results of hierarchal regression.

Variable	Voice		Self-efficacy		Psychological safety	
			
	Model 1	Model 2	Model 3	Model4	Model 5	Model 6
***Control variables***						
Leaders’ age	–0.01	–0.10	0.05	0.05	–0.05	–0.07
Leaders’ gender	0.15	0.13	0.01	0.02	0.07	0.09
Employees’ age	0.22*	0.21**	0.09	0.07	–0.00	–0.02
Employees’ gender	0.04	0.04	0.04	0.03	–0.02	–0.02
Dyadic tenure	0.04	0.04	–0.04	–0.02	0.02	0.06
***Independent variable***						
Leader humility (LH)	0.15*	–0.05	0.41**	0.31**	0.60**	0.35**
***Mediator***						
Self-efficacy		0.15**				
Psychological safety		0.22**				
***Moderator***						
Manipulative intention (MI)				–0.23**		–0.43**
***Interaction***						
LH × MI				–0.19**		–0.15**
*R* ^2^	0.09	0.15**	0.18**	0.21**	0.38**	0.45**

*p < 0.05, **p < 0.01.

With 20,000 Monte Carlo replications, we presented the tests on the mediation relationships posited in Hypothesis 2 and 3. The result revealed that psychological safety played a mediating role in the relationship between leader-expressed humility and voice [indirect effect = 0.13, 95% CI = (0.060, 0.266)]. In addition, self-efficacy played a mediating role in the relationship between leader-expressed humility and voice [indirect effect = 0.06, 95% CI = (0.008, 0.138)]. Thus, Hypothesis 2 and 3 were supported.

**FIGURE 2:**
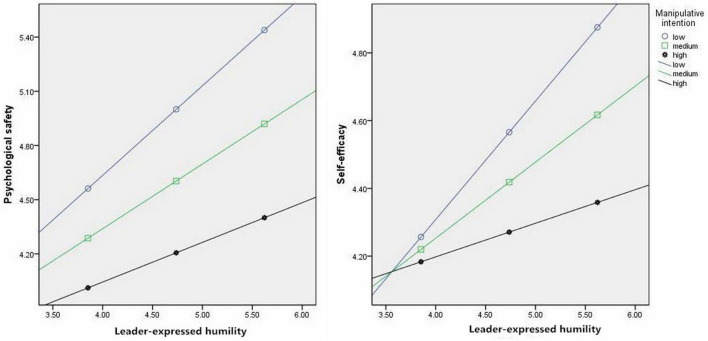
Moderating effect of perceived manipulative intention.

Table 4 showed that the interaction between leader-expressed humility and perceived manipulative intention had a predictive effect on self-efficacy (Model 4: β = –0.19, *p* < 0.01) and psychological safety (Model 6: β = –0.15, *p* < 0.01). Moreover, perceived manipulative intention was divided into high, medium, and low groups according to the mean ± SD to plot the interaction effect. As illustrated in Figure 2 increases in perceived manipulative intention weakened the positive relationship between leader-expressed humility and psychological safety and the positive relationship between leader-expressed humility and self-efficacy. Thus, Hypothesis 4 and 5 were supported.

With 20,000 Monte Carlo replications, we presented the tests on the moderated mediation relationships posited in Hypothesis 6 and 7. We found that the indirect effect of leader-expressed humility on voice via psychological safety is more positive when perceived manipulative intention is lower [–1SD below mean; indirect effect = 0.13, 95% CI (0.047, 0.231)] than when it is higher [+ 1 SD above mean; indirect effect = 0.06, 95% CI (0.007, 0.123)]. The difference between high and low for the indirect path via psychological safety was significant [indirect effect difference = –0.07, 95% CI (–0.139, –0.021)]. In line with our own expectations, the indirect effect of leader-expressed humility on voice via self-efficacy was more positive when perceived manipulative intention was lower [–1SD below mean; indirect effect = 0.09, 95% CI (0.009, 0.171)] than when it was higher [+ 1 SD above mean; indirect effect = 0.02, 95% CI (–0.008, 0.065)]. The difference between high and low for the indirect path via self-efficacy is significant [indirect effect difference = –0.07, 95% CI (–0.146, –0.005)]. Thus, Hypothesis 6 and 7 are supported.

## Discussion

### Summary

This study demonstrated the effect of leader-expressed humility on employees’ voice through psychological safety and self-efficacy. Moreover, drawing on attribution theory, we found that perceived manipulative intention negatively moderated the indirect effect of leader-expressed humility on employee voice through psychological safety and self-efficacy.

### Theoretical implications

According to Morrison’s model of employee voice (Morrison, 2011), it is more comprehensive to explore the effects of leader-expressed humility on employees’ voice behavior from the dual path of employees’ perceived safety and efficacy. To our knowledge, there is no study on humble leadership and voice has incorporated dual mediators, such as psychological safety and self-efficacy (Wei et al., 2015; Lee et al., 2017). By employing psychological safety and self-efficacy as mediators, this study enriches the comprehensive understanding the impacts of leader-expressed humility on employees’ voice behavior.

In addition, Owens and Hekman (2012) indicated that leader humility is effective only if employees perceive their leader to be sincere and authentic, but studies on leader humility have not addressed this assertion. According to Martinko et al. (2007, 2011), more attention should be paid to attribution theory in the organizational sciences, and especially to relating attributions to leadership. As leaders reap the benefits from their employees’ good performance, employees may be suspicious of their leader’s intentions (Grant and Hofmann, 2011). Attribution theory was applied in this study to explore the possible negative effects of leader-expressed humble behaviors when these behaviors are perceived as manipulative by their employees. By examining whether attributing manipulative intention to leader-expressed humility has an influence on the employee’s voice behavior, this research finds that perceived manipulative intention caused employees to doubt their leader’s humble behavior, thereby strengthening their defensive mindset against their leader and weakening the positive effect of leader-expressed humility. The theoretical rationale and empirical findings in this study contribute to the literature on leader humility by emphasizing the importance of employees’ attribution. This study extended the research on the boundary conditions of leader-expressed humility’s positive effects and reminded that we need to focus more on the boundary conditions of leader-expressed humility. Our investigation thus presents a more nuanced picture showing how employees’ perceptions of leader-express humility can be undermined by their manipulative intention attributions.

### Practical implications

This study provides leaders with a new approach to being receptive toward employee ideas and concerns. As expressed humility can be acquired (Argandona, 2015), enterprises should offer training courses to encourage leaders to be humble. Appropriate human resource policies that highlight the importance of virtues including selfless humility should be designed by organizations. At the practical level, this study provides an important basis for HR managers that oversee recruiting managers and leadership development programs. Leaders need to take the initiative to eliminate power barriers, break hierarchical conventions, and establish respect and trust with employees to create a safe psychological climate for employees (Edmondson, 1999; Kiazad et al., 2010).

In addition, although leader-expressed humility can promote employee voice behavior, the effect varies depending on perceived manipulative intention. Sincerity is a prerequisite for such bottom-up leadership behavior in an organization. Humility entails being honest with yourself and open to others rather than spurious acts of humility (Chiu and Hung, 2020). Leaders should be aware of the level of congruence between their expressed behaviors. The attributive lenses through which their employees interpret those behaviors are equally important. Leaders should act for the benefit of the group and uphold the value of employees prevent employees from labeling the leader as pseudo humble (Dasborough and Ashkanasy, 2002).

### Research limitations and suggestions

There were several limitations in this study. First, although we collected data from a two-wave and multi-source survey, such a research design maybe also constrained on causality. Future research could conduct a longitudinal research to confirm the causality proposed by this research. Second, our study samples were collected from China. Specifically, because Chinese culture traditionally encourages humility, Chinese leaders engage in humble behaviors more frequently than Western leaders do (Hu et al., 2018). Future studies can study populations of a different culture to explore cross-cultural differences in both the effects of leader humility and how leader humility is performed. Third, this study didn’t explore individual difference, such as gender and education, however, most of the leaders investigated in this study were female (71%). The relationship between gender difference and leadership style has attracted the attention of researchers. For example, studies have found significant differences in effectiveness in terms of transformational leadership between men and women (Kent et al., 2010). Due to the predominance of female leaders in this study, future studies should consider humble leadership effectiveness from the perspective of gender difference. Fourth, considering the manipulation perceived by workers, an additional component could influence the model, the emotions felt by workers toward the leader (fear, awe, anger). Studies have indicated that pseudo-transformational leadership increases subordinates’ perceptions of fear, insecurity, and abusive supervision in the workplace (Christie et al., 2011). Hence, future studies could explore the effects of emotions elicited by pseudo-humility (D’Errico, 2019). Fifth, despite taking precautions to reduce social desirability bias, it is not possible to completely eliminate its effects. Future studies should make social desirability a control variable to mitigate the social desirability bias. Finally, power analysis was not conducted in this study. Future studies should conduct power analysis to evaluate the sample size.

## Data availability statement

The raw data supporting the conclusions of this article will be made available by the authors, without undue reservation.

## Author contributions

W-QZ was responsible for data collection, writing the manuscript, and conceptualizing the models. S-CC acted as the Principal Investigator and oversaw the study from its inception to completion. Both authors contributed to the article and approved the submitted version.
